# Audience spontaneous entrainment during the collective enjoyment of live performances: physiological and behavioral measurements

**DOI:** 10.1038/s41598-020-60832-7

**Published:** 2020-03-02

**Authors:** Martina Ardizzi, Marta Calbi, Simona Tavaglione, Maria Alessandra Umiltà, Vittorio Gallese

**Affiliations:** 1 Unit of Neuroscience, Department of Medicine and Surgery, University of Parma, Parma, Italy; 2 Department of Food and Drug, University of Parma, Parma, Italy; 3 Department of Art History Columbia University, Italian Academy for Advanced Studies, Columbia University, New York, NY USA

**Keywords:** Emotion, Psychology

## Abstract

Cardiac synchrony is a crucial component of shared experiences, considered as an objective measure of emotional processes accompanying empathic interactions. No study has investigated whether cardiac synchrony among people engaged in collective situations links to the individual emotional evaluation of the shared experience. We investigated theatrical live performances as collective experiences evoking strong emotional engagement in the audience. Cross Recurrence Quantification Analysis was applied to obtain the cardiac synchrony of twelve spectators’ quartets attending to two live acting performances. This physiological measure was then correlated with spectators’ emotional intensity ratings. Results showed an expected increment in synchrony among people belonging to the same quartet during both performances attendance and rest periods. Furthermore, participants’ cardiac synchrony was found to be correlated with audience’s convergence in the explicit emotional evaluation of the performances they attended to. These findings demonstrate that the mere co-presence of other people sharing a common experience is enough for cardiac synchrony to occur spontaneously and that it increases in function of a shared and coherent explicit emotional experience.

## Introduction

Collective experiences are ubiquitous aspects of human culture where emotional activity finds its breeding epicenter. Being involved in collective events, like religious rituals, parades or team sports, fosters prosociality^1^ and contributes to the creation of emotional bonds among group members^2^. Coherently, it has been shown that the presence of others modulates how individuals feel, express and perceive emotions^3^.

Amongst the various group experiences populating our social life, specific forms of “collective art” can be included. Indeed, even if some forms of art can be described as “individual”, in the sense that usually people enjoy them alone, others are commonly appreciated together with other people. This is the case of several performative arts (e.g., theatre, dance, music) in which people share the artistic experience as part of a group. Studies show that people react differently to these forms of art if they enjoy them alone or with others, suggesting that the presence of others plays a role. For example, at the autonomic level, people react more to music when they are alone than when are in a group^4,5^. Differently, watching an aggressive movie clip modulates spectator’s subsequent behaviors, on the basis of the attitude of the co-spectators^6^. Furthermore, how people react to the mere presence of others influences their parasympathetic response to shared filmic experience^7^. One interesting component of collective experiences, that recently drew increasing attention, is the spontaneous synchronization among group members. Synchrony is a well-known phenomenon occurring at different levels (e.g., behavioral, physiological and neural), and it is related to the presence of similar reactions among group members within a short period of time. For example, people spontaneously synchronize their body movements when engaged in a conversation^8,9^, and these coordinated movements contribute to observers’ perception of affiliation^10^. Similarly, verbal synchrony in large groups produces affiliation feelings and increases group members’ coordinative efforts^11^. Furthermore, inter-brain synchrony has been found associated with coordination of speech rhythm in human-to-human social interactions^12^. Interestingly, being in a group performing a simple motor task is enough to induce spontaneous behavioral and physiological group synchrony^13^. In the context of collective forms of art, studies demonstrate that also during these peculiar shared experiences synchrony occurs. Indeed, the members of a chorus synchronize their cardiorespiratory activity when singing^14^, as well as, clapping spectators synchronize their hand movements after a showstopper^15^. More recently, Bernardi and colleagues^16^ find enhanced autonomic synchrony among passive spectators while listening to organ music excerpts.

Synchrony, especially at the physiological level, is a component of shared experiences potentially providing an objective measure of internal processes accompanying empathic interactions^17^. Indeed, research in social psychology demonstrates that synchrony spontaneously occurs among people, even when not directly interacting^18^, and it fosters feelings of togetherness, similarity, cooperation, and social bonding^19–21^. Interestingly, high levels of behavioral and physiological synchrony among people influence how they emotionally rate different shared activities. For example, people physiologically synchronized during digital multiplayer game attribute higher self-reported ratings of affective involvement to this recreational social interaction^22^. During dyadic relations, it has been demonstrated that the accuracy in the rating of other’s negative affect intensity are uniquely associated with measures of the dyad’s physiological synchrony^23^. Furthermore, perceived emotional engagement is consistently linked with high synchrony of dyads’ skin conductance responses^24^. Also spontaneous behavioral synchronization between two strangers predicts the individual ratings of positive affect intensity attributed to the shared social interaction^25^. All these results show how individual physiological signals can mutually react and synchronize during different kind of shared experiences, and how the extent of such synchrony is indicative of people explicit evaluation of emotional intensity. This link is only partially addressed in the context of collective forms of art. For example, non-expert listeners can spontaneously perceive the patterns of coordination that musicians create together when improvising along a backing track; this perception influences listeners’ explicit appreciation of the recordings aesthetic features^26^. Furthermore, the occurrence of spectators’ synchronized behaviors and physiological reactions is linked to the presence of emotional and aesthetic highlights in a movie^27,28^. Despite these supporting results, no study has directly investigated the potential link between spectators’ synchrony and their explicit ratings of the emotional intensity attributed to the shared artistic experience. This empirical question could attract widespread interest considering that from a phylogenetic perspective, which endorses biological and cultural continuity, the increased individual feelings of emotional intensity associated with behavioral and physiological synchrony are considered the ancestral drivers of the emergence of primitive and modern collective practices which foster the evolution of complex human societies^29^.

With the present study, we aimed to investigate and explore the role of spectators’ synchrony in the context of collective forms of art. Specifically, we studied the potential increment in cardiac synchrony among spectators of the same live performances and how this spontaneous physiological synchrony could be related to spectators individual rating of performance emotional intensity. Among the different levels at which synchrony can occur, cardiac synchrony is our primary candidate to be investigated, because autonomic responses are key substrates of emotional experience^30–32^. Moreover, spontanous autonomic reactions are extremely common in responses to art^33–35^. To accomplish our goal, we recruited 12 quartets of spectators, each of which attended two live performances (monologues) played by one out of 12 actors. For each participant we calculated her/his mean cardiac synchrony with the other members of the quartet (in-group synchrony) and with three randomly chosen spectators belonging to different quartets (out-group synchrony). These measures were recorded before and during attendance to performances. At the end of the acting session, explicit evaluation of monologues emotional intensity was rated by all spectators.

If the hypothesized cardiac synchrony among spectators enjoying the same performance occurred, we would expect higher level of in-group synchrony with respect to out-group synchrony. Furthermore, if audience’s cardiac synchrony was associated with the rated emotional intensity of monologues, a significant correlation between the degree of in-group synchrony and the explicit emotional rating is predicted.

## Results

Due to technical problems in the data acquisition, one quartet of spectators was excluded from the analyses; then 44 spectators composed the total sample of 11 quartets.

### Behavioral results

Participants’ explicit ratings were entered into a MANOVA, with Monologue (i.e., November 20^th^, August 10^th^) as within-subjects factor. A non-significant Box’s M test (p = 0.211) indicated homogeneity of covariance matrices of the dependent variables across the monologues. The multivariate effect was not significant by Monologue (*Pillai’s trace* = 0.19; F_(10,71)_ = 1.61, p = 0.121, partial η^2^_p_ = 0.19), indicating absence of difference in participants’ explicit ratings between monologues.

Results of Principal Component Analysis (PCA) conducted on the ratings of the monologue November 20^th^ was found to be suitable according to the correlation matrix, overall Kaiser–Meyer–Olkin (0.75) and Bartlett’s test of sphericity (P < 0.001). The PCA revealed three components that had eigenvalues greater than one (component 1^Nov20^: 4.55, component 2^Nov20^: 1.47, component 3^Nov20^: 1.1). This three-components solution explained 71.25% of the total variance (component 1^Nov20^: 45.49%, component 2^Nov20^: 14.86%, component 3^Nov20^: 10.91%). The first component included the rating about the emotional intensity of the performance together with the rating of anger intensity in text and performance, the rating about emotional engagement and the overall quality of the performance. The second component indicated a considerable commonality in the variability for ratings about sadness intensity both in text and performance and text general emotional intensity. The third component combined the ratings about fear intensity in text and performance.

Results of PCA conducted on the ratings about the monologue August 10^th^ was found to be suitable according to the correlation matrix, overall Kaiser–Meyer–Olkin (0.75) and Bartlett’s test of sphericity (P < 0.001). Again, the PCA revealed three components that had eigenvalues greater than one (component 1^Aug10^: 4.9, component 2^Aug10^: 1.5, component 3^Aug10^: 1.02). These three components explained 74.5% of the total variance (component 1^Aug10^: 49.12%, component 2^Aug10^: 15.12%, component 3^Aug10^: 10.25%).

Also in this case, the first component included the rating about the emotional intensity of the performance together with the explicit ratings of sadness in text and performance and the ratings about emotional engagement and quality of the performance. The second component indicated a considerable commonality in the variability of ratings about anger intensity both in text and performance and in text emotional intensity. The third cluster, as in November 20^th^, showed the ratings about fear intensity both for text and performance.

**Figure 1:**
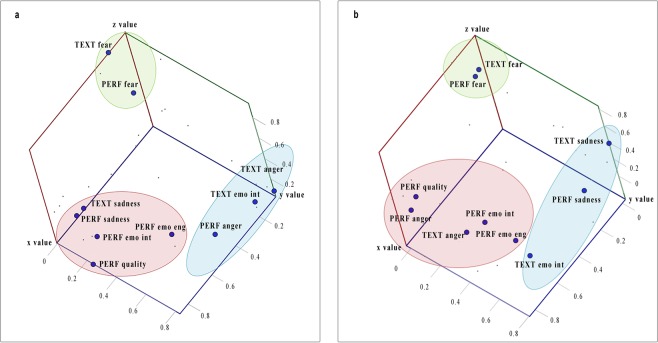
Results of Principal Component Analyses conducted on the explicit ratings of the two monologues. Panel a: November 20^th^; Panel b: August 10^th^. Red = component 1; light blue = component 2; green = component 3. **PERF**:in performance; **TEXT:** in text **fear =** Fear intensity**; anger =** Anger intensity**; sadness =** Sadness intensity**; emo int =** Emotional intensity; **quality =** Performance Quality**; emo eng =** Emotional involvement.

Please, refer to Fig. 1 for a graphical representation of the three components obtained for the two monologues. See Table 1 for the rotated component matrices from the PCA conducted on the explicit ratings of the two monologues.

**Table 1:** Rotated component matrices from the Principal Component Analyses for the explicit rating of the monologues.

	Anger intensity in Performance	Performance Quality	Anger Intensity in Text	Emotional intensity of performance	Emotional involvement
Component 1 November 20th	0.852	0.772	0.689	0.687	0.612
	Sadness intensity in text	Sadness intensity in performance	Emotional intensity of text		
Component 2 November 20th	0.804	0.744	0.579		
	Fear intensity in performance	Fear intensity in text			
Component 3 November 20th	0.841	0.834			
	Performance Quality	Emotional intensity of performance	Sadness Intensity in Performance	Sadness intensity in text	Emotional involvement
Component 1 August 10th	0.868	0.824	0.781	0.743	0.566
	Anger intensity in text	Emotional intensity of text	Anger intensity in performance		
Component 2 August 10th	0.830	0.783	0.7		
	Fear intensity in text	Fear intensity in performance			
Component 3 August 10th	0.941	0.859			

### Results of Cross Recurrence Quantification Analysis

First, the metrics derived by the Cross Recurrence Quantification Analysis (CRQA) conducted on the three rest periods (i.e., baseline, rest1 and rest2) were entered into a MANOVA with Period (i.e., Baseline, Rest1 and Rest2) and Group (i.e., In-group; Out-group) as within-subjects factors. This analysis was performed to assure that no significant leftovers existed between monologues. A non-significant Box’s M test (p = 0.194) indicated homogeneity of covariance matrices of the dependent variables across the resting periods. The multivariate effect was not significant by Period (*Pillai’s trace* = 0.04; F_(8,464)_ = 1.05, p = 0.394, partial η^2^_p_ = 0.02). Interestingly, we found a significant multivariate effect by Group (*Pillai’s trace* = 0.11; F_(4,231)_ = 7.06, p < 0.0001, partial η^2^_p_ = 0.11). Univariate tests showed that there were significant differences across the conditions for percentage recurrence (In-group = 0.20, SE 0.002; Out-group = 0.18, SE 0.002; F_(1,234)_ = 15.53, p < 0.0001, partial η^2^_p_ = 0.06), entropy (In-group = 0.65, SE 0.005; Out-group = 0.63, SE 0.005; F_(1,234)_ = 5.07, p = 0.025, partial η^2^_p_ = 0.02) and max diagonal length (In-group = 44, SE 0.595; Out-group = 40.43, SE 0.595; F_(1,234)_ = 17.98, p < 0.0001, partial η^2^_p_ = 0.07).

**Figure 2:**
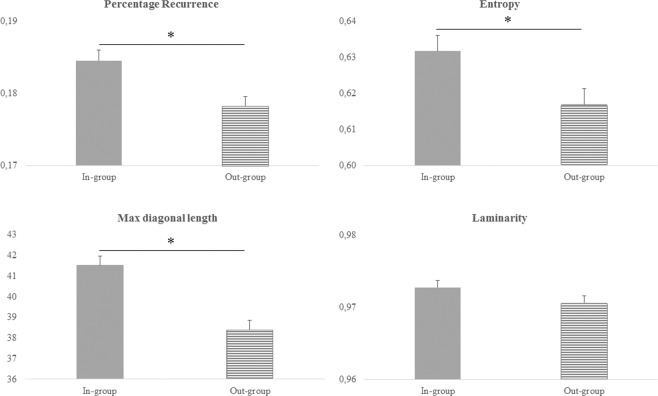
Differences in percentage recurrence, entropy and max diagonal length between in-group and out-group. Error bars depicted SE, * = p < 0.05.

To test our main hypothesis, the metrics obtained from the CRQA conducted on the monologues were compared in a MANOVA with Condition (i.e., November 20^th^, August 10^th^), Group (i.e., In-group, Out-group) and Time (i.e., T1, T2) as within-subjects factors. A non-significant Box’s M test (p = 0.115) indicated homogeneity of covariance matrices of the dependent variables across the conditions. The multivariate effects of Group (*Pillai’s trace* = 0.09; F_(4,341)_ = 8.52, p < 0.0001, partial η^2^_p_ = 0.09) (Fig. 2) and Time (*Pillai’s trace* = 0.1; F_(4,341)_ = 9.05, p < 0.0001, partial η^2^_p_ = 0.1) were significant.

Univariate tests showed that there were significant differences across in-group and out-group for percentage recurrence (In-group = 0.19, SE 0.001; Out-group = 0.18, SE 0.001; F_(1,344)_ = 10.02, p = 0.002, partial η^2^_p_ = 0.03), entropy (In-group = 0.63, SE 0.004; Out-group = 0.62, SE 0.004; F_(1,344)_ = 5.60, p = 0.019, partial η^2^_p_ = 0.02) and max diagonal length (In-group = 41.53, SE 0.45; Out-group = 38.41, SE 0.45; F_(1,344)_ = 23.98, p < 0.0001, partial η^2^_p_ = 0.07). Furthermore, univariate analyses showed that there were significant differences across the first two minutes and the last two minutes only for entropy (T1 = 0.61, SE 0.004; T2 = 0.64, SE 0.004; F_(1,344)_ = 32.18, p < 0.0001, partial η^2^_p_ = 0.09).

### Correlations between explicit classification of monologues emotional intensity and CRQA metrics

Contrary to our expectations, Bonferroni corrected Pearson’s correlation analyses between monologues emotional intensity ratings and CRQA metrics did not show any significant result (all p_s_> 0.45).

In-group convergence and out-group convergence scores were calculated. Note that High in-group convergence score corresponds to great participants’ deviation in the rating compared to spectators belonging to participants’ quartet. On the contrary, low in-group convergence score corresponds to low participants’ rating deviation from in-group members’ scores.

**Figure 3:**
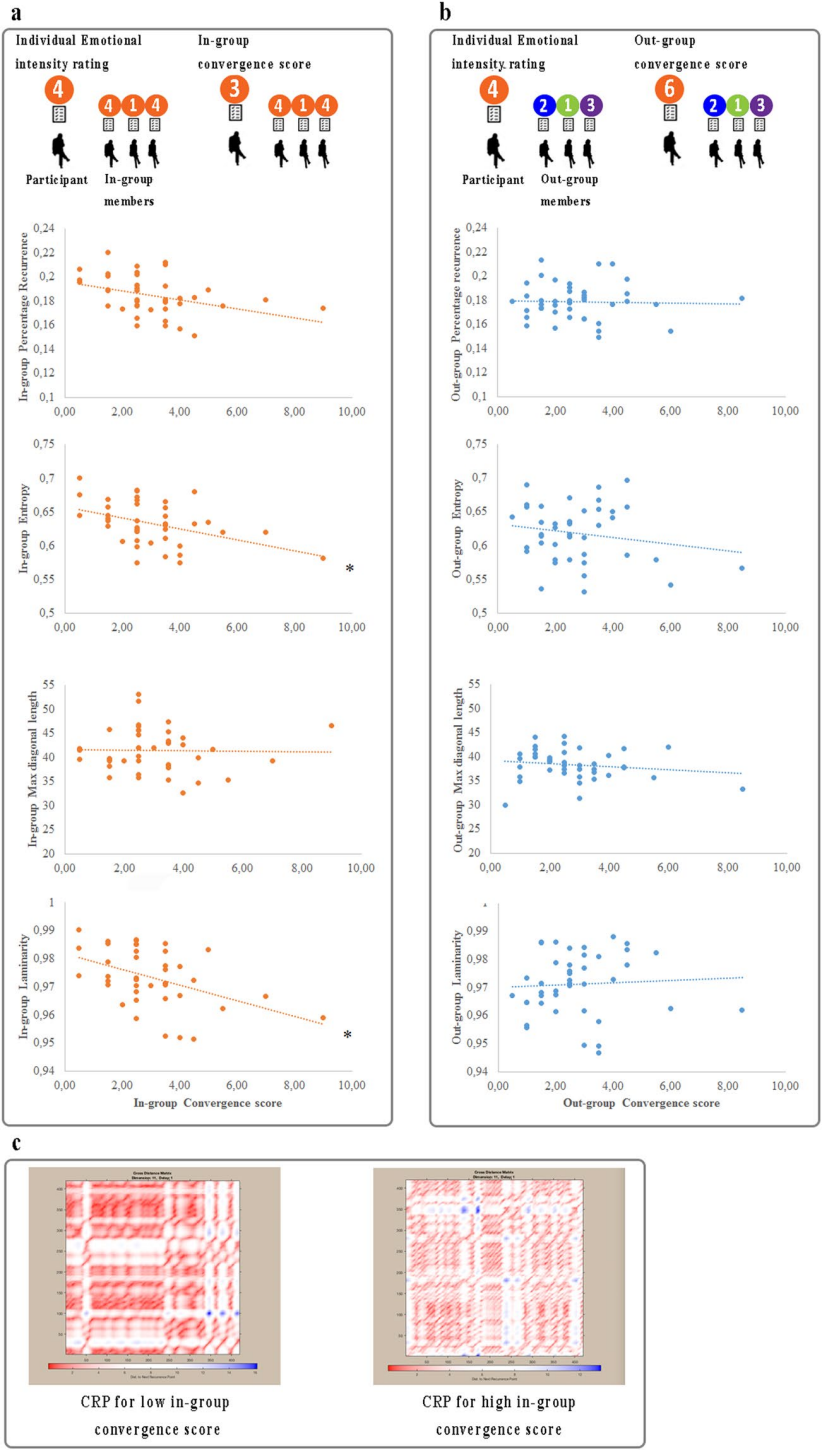
Panel a: correlation plots between participants’ mean in-group CRQA metrics and in-group convergence scores; Panel b: correlation plots between participants’ mean out-group CRQA metrics and out-group convergence scores; Panel c: exemplificative cross recurrence plots (CRP) for high and low in-group convergent participants. Low in-group convergence score corresponds to low participant’s rating deviation from in-group members’ scores. *Bonferroni corrected p < 0. 0125.

Interestingly, results showed significant inverse correlations between in-group convergence score and in-group metrics of laminarity (r_42_ = −0.45, Bonferroni corrected p = 0.003, two-tailed) and entropy (r_42_ = −0.43, Bonferroni corrected p = 0.005, two-tailed) (Fig. 3, panel a). A near to significant inverse correlation was found between in-group convergence score and in-group percentage recurrence (r_42_ = −0.38, Bonferroni corrected p = 0.014, two-tailed) (Fig. 3, panel a). No significant correlations were found between participants’ mean out-group CRQA metrics and out-group convergence scores (all p_s_ > 0.24) (Fig. 3, panel b).

## Discussion

The present study aimed to investigate the potential increment in cardiac synchrony among spectators of the same live performances and to study how this spontaneous physiological synchronization could be related to the explicit emotional intensity ratings attributed to the performances collectively enjoyed.

Participants rated the monologues played by the actors according to the emotional intensity they perceived in the performances, together with other qualities of the two acting sets. This information was included in a principal component analysis in order to better qualify the features related to the explicit evaluation of the emotional intensity of the performances. Interestingly, the principal component analysis showed that the emotional intensity of the monologue November 20^th^ was mainly related to the anger intensity perceived both in the text and in the performance, as well as to the overall quality of the performance and spectators’ engagement (component 1^nov20^). Differently, the rating of the emotional intensity of the monologue August 10^th^ was associated to the amount of sadness perceived both in the text and in the performance and, again, to the general performance quality and audience’s engagement (component 1^aug10^). These results exemplify the distinctiveness and the intrinsic ambiguity of performative arts. Indeed, the two monologues did not differ in the absolute amount of conveyed emotional intensity, but rather in the way the emotional intensity was related to other features of the monologue.

As expected, an increment in cardiac synchrony among the members of the same audience group, when compared with out-group members, was found during both the rest periods and monologue sessions. The increase in the in-group cardiac synchrony already clearly visible in the rest periods is in line with several studies showing the relevance of basic sensory interpersonal linkages in behavioral and neurophysiological synchrony. At the behavioral level, unintentional interpersonal coordination emerges when visual information about the other person is available without any additional effect of direct verbal interaction^36–38^. Similarly, at the physiological level, the mere co-presence of other people sharing a common experience in the absence of a direct face-to-face interaction produces higher levels of cardiac synchrony^18^. Also, brain-to-brain synchrony between strangers correlates with the presence of non-verbal signals rather than with direct verbal interactions^39^. These results suggest that the mere co-presence of other people sharing a common experience is the minimal condition necessary for synchrony to occur at different levels.

Such higher in-group cardiac synchrony, when compared with out-group cardiac synchrony, was also found during monologues attendance. This result enriches the literature about the collective forms of art^16^, showing how spontaneous cardiac synchrony occurs automatically among the spectators of the same theatrical performance even if they are not directly interacting with each other. Again, this result further stresses the importance of the actual co-presence of spectators. Indeed, to be synchronized it is not enough to enjoy the same show played by different actors at different times. Differently, it seems that people have to share the same experience at the same time to significantly increase their cardiac synchrony. Coherently with another study conducted on a cohort of students attending the same lesson^40^, greater synchrony among spectators may indicate an actual joint attention state when the audience is collectively awaiting or enjoying the same live performance.

Interestingly, in-group cardiac synchrony did not correlate with the perceived *emotional intensity* of the performances, as expected. Conversely, it correlated with the degree of in-group *convergence* on the emotional evaluation of the performances. In other words, in-group cardiac synchrony did not correlate with participants’ individual evaluation, but with participants’ degree of convergence with the other group members on their explicit evaluation. In contrast, out-group convergence score was not related to out-group level of cardiac synchrony. This negative result confirms the specificity of the relation between the actual collective enjoyment, the increment in cardiac synchrony and the convergence on explicit evaluation.

Recently, research on audiences of live performances has gained interest in part because group of spectators provide a valid and ecological setting for investigate group dynamics during collective experiences. In particular, physiological synchrony between audience members and performers has been found to be influenced by spectators’ artistic preference^41^, subjective attentional modulation^42^ and intersubjective engagement^43^. Similarly, spectators’ enjoyment during a live dance performance seems to be sensitive to how dancers coordinate and synchronized their movements, rather than how much they move^33^. All these studies confirm how spectating is not simply sitting and being automatically “moved” by the artwork. Rather, it is an active attitude easily influenced by the nature of the relationship between the spectator and the performer. Our results extend this statement supporting the relevance of the dynamics occurring among the members of an audience group.

Some limitations of the present study should be highlighted. The extended literature about physiological synchrony reveals how context is a key factor especially when dyads of strangers are considered^17^. Synchrony has been found to differ across contexts, indicating that participants who are in the same setting display physiological synchrony in some contexts but not in others. This context dependency can not be neglected especially when synchrony is interpreted in relation with other psychological variables and in specific conditions, as in our case. Considering the specific context here tested where participants were informed to be the audience of two live performances, we can not rule out that different contexts - like an actual interaction between people - would evoke stronger emotional engagement and potentially greater cardiac synchrony. Moreover, some methodological choices further limit the general validity of our conclusions. First, in the attempt to combine empirical rigor with ecological context, the setting tested here did not mimic exactly a conventional theatrical experience. Second, the synchronization between actor and spectator could not be investigated, and third, the two selected monologues had both a negative emotional valence.

In conclusion, the present results suggest that enjoying together theatrical performances increases spectators’ physiological synchrony in function of a shared and coherent emotional experience. It seems that the emotional experience of collective forms of art may be collective at least at two related levels: (1) the convergence of explicit evaluation, and (2) the physiological synchrony. According to phylogenetic theories about the development of aesthetic experience in humans, the adaptive advantage of art might be associated to the promotion of group cohesion^44^. Considering aesthetic experience as a multifaceted phenomenon involving perceptual, affective and cognitive processes^45,46^, further research should investigate whether group convergence, and the related increased cardiac synchrony, may play a role in the aesthetic experience of collective forms of art. Indeed, spectators’ synchrony and judgment convergence may be valid precursors of specific collective outcomes of art experience^45^. Our results suggest that in order to better understand how collective art works, one should move from a single-person to a “multi-person” approach, focusing on the interpersonal dynamics of aesthetic experience, rather than on individual responses.

## Materials and Methods

The experimental protocol was approved by the Institutional Review Board of the University of Parma and it was in line with the Declaration of Helsinki, 2013. The study general purposes and procedures were explained to participants. After participant’s agreement to participate in the study, a written informed consent was collected. The experiment was carried out as part of the project “Habeas Corpus” aimed at merging neuroscience with dramatic performative arts, organized and supported by Fondazione Prada. The experiment took place in Venice, Ca Corner della Regina, where a quiet, isolated and soft illuminated hall was dedicated to the experiment.

### Participants

Participants were healthy volunteers enrolled as spectators in the study. Forty-eight (male = 24, female = 24) spectators participated in the study. The number of participants involved in the present study exeeded the a priori required total sample size (n. 44) estimated by means of statistical power analysis (a priori sample size n. evaluated for 1 − β = 0.95, α  =  0.05 and effect size  =  0.25). They were aged between 21 to 49 years, with mean age of 30 years (SE = 0.99). The national composition of the sample was mixed (64.6% Italian, 12.5% English, 4.2% American, 4.2% Polish, 4.2% French, 2.1% Portuguese, 2.1% Serbian and 6.3% did not provide this information), all of them had a good or excellent level of spoken and written English. Overall, participants regularly watched movies and attended to theatre performances (mean of films watched in a month = 8.29, SE = 1.17; mean theatre performances attended in a month = 1.14, SE = 0.19) and almost half of them had a previous experience as actor (no experience = 43.8%, experience = 54.2%). Lastly, most of participants had good theatrical and filmic knowledge (filmic knowledge: none 6.3%; sufficient 12.5%; good 58.3%; excellent 20.8%; Theatrical knowledge: none 8.3%; sufficient 41.7%; good 47.9%; excellent 2.1%).

### Procedure

The experiment involved 12 professional actors and 48 spectators. Each of the twelve actors (6 males, 6 females) performed two monologues, while they were seated on a bench in front of four spectators (2 males, 2 females) sitting on an opposite bench. Spectators’ bench was located two metres far from the actor’s one. Each actor was assigned to a different audience group. Monologues order was balanced among groups. Spectators were instructed to not interact neither with the actor nor with the other members of the audience group. The actors were advised to play the monologues in front of the audience avoiding any other direct interaction with the spectators. Actors worked with a professional director to prepare their performances, so all actors’ performances were based on the same monologues text but played differently according to the individual disposition of each actor. Consequently, the performances had different durations (mean 5.41′, SD 1.26′). Before the beginning of the first monologue and after the end of each monologue, two minutes of Electrocardiogram (ECG) at rest were recorded, for a total of three rest periods in the entire session. During the rest periods, actor and spectators sat quietly without interacting with each other. The first ECG rest period was recorded as baseline parameter, whereas the two post-monologue ECG rest periods were recorded in order to verify the presence of potential residual physiological effects of the performances on subsequent periods. All the actors weared the same dark color clothes designed by Prada.

The monologues performed by the actors were inspired by the infamous story of a young man guilty of a school shooting. One monologue (“August 10^th^”) was taken from the online journal of this individual: it consists of a self-reflective monologue about his internal states. The second monologue (“November 20^th^”) was a first-person narrative by another author of the bullying that had tormented this individual during his childhood and adolescence. All participants, before entering the experimental session, read a brief pamphlet containing the story of the young man, the texts of the two monologues and some relevant contextual information. This procedure was followed to assure that all participants had the same information about the monologues that they were going to attend. Full monologue text are available from the corresponding author on reasonable request. Live performances were selected, instead of their recorded version, because they produce greater audience engagement^47^.

**Figure 4:**
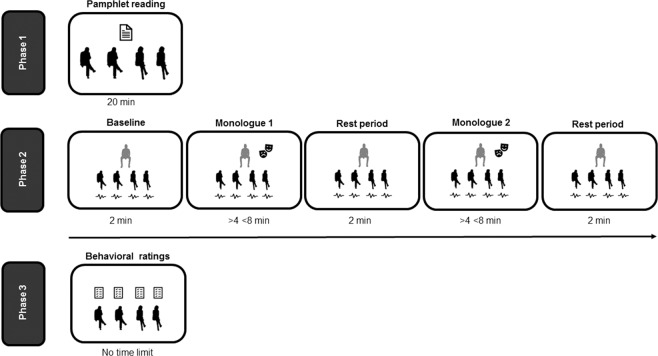
Experimental procedure.

At the end of the acting session, spectators filled questionnaires in which they explicitated their personal evaluations of the performances just attended. All questions were rated on a Likert scale ranging from 1 to 5. For each monologue, participants rated the emotional intensity of the performances toghether with other extra emotional qualities of the monologues. These additional questions were: how much emotionally intense was the *text* of the monologues; how much intense were the negative emotions (i.e., anger, fear, and sadness) conveyed by both the texts and performances; how much participants felt emotionally involved during the performance and how they judged the overall quality of the performance. The 3 negative emotions investigated in the questionnaire were chosen on the basis of the literature^48^ and thanks to a stylistic analysis of monologue texts done by the director. This information was collected to have a detailed qualitative description of the two monologues, and specifically to better understand what emotional features were related to the evaluation of the emotional intensity of the performance (see above, principal component analyses). Furthermore, participants filled the Behavioural Inhibition System and Behavioural Activation System questionnaire^49^, the Interpersonal Reactivity Index^50^ and the Toronto Alexithymia Scale^51^, in order provide a description of the personality traits of our sample of participants. Please refer to Fig. 4 for a graphical representation of the procedure and to Table 2 for participants’ mean ratings and questionnaire scores.

**Table 2:** Mean and Standard Error (SE) of participants’ explicit ratings and questionnaire scores.

		Mean	SE
Emotional intensity of performance	AUG10	3.34	0.15
	NOV20	3.68	0.15
Anger intensity in Performance	AUG10	2.94	0.15
	NOV20	3.32	0.15
Fear intensity in Performance	AUG10	2.80	0.16
	NOV20	2.93	0.16
Sadness intensity in performance	AUG10	3.61	0.14
	NOV20	3.56	0.14
Emotional intensity of text	AUG10	3.20	0.15
	NOV20	3.73	0.15
Anger Intensity in Text	AUG10	3.22	0.15
	NOV20	3.63	0.15
Fear intensity in text	AUG10	2.83	0.17
	NOV20	3.10	0.17
Sadness intensity in text	AUG10	3.71	0.16
	NOV20	3.78	0.16
Emotional involvement	AUG10	2.82	0.15
	NOV20	3.12	0.15
Performance Quality	AUG10	3.78	0.14
	NOV20	3.76	0.14
Interpersonal Reactivity Index	Empathic concern	19.13	4.28
	Personal distress	10.79	7.42
	Perspective taking	17.18	5.21
	Fantasy scale	18.67	4.75
Toronto Alexithymia Scale	Difficulty Describing Feelings	12.97	4.61
	Difficulty Identifying Feeling	17.77	5.88
	Externally Oriented Thinking	15.64	4.06
	Tot	46.39	11.54
Behavioral Inhibition System and Behavioral Activation System questionnaire	BIS	23	3.75
	BAS	45.85	7.55
	BAS-drive	13.12	2.80
	BAS-fun seeking	13.25	3.36
	BAS-reward responsiveness	19.47	3.81

AUG10 = Monologue August 10^th^; NOV20 = Monologue November 20^th^.

### Data analysis

#### Behavioral measures

Explicit ratings of all participants were entered into a MANOVA, with Monologue (i.e., November 20^th^, August 10^th^) as within-subjects factor. The same ratings were used to compute Principal Component Analysis (PCA), as implemented in IBM SPSS statistics 23, to investigate what kind of monologue quality was more related to the evaluation of the performance emotional intensity. PCA was performed, separately for the two monologues, in a matrix containing all the behavioral measures (10 ratings by 44 participants). The suitability of each PCA was assessed prior to analysis by inspection of the correlation matrix (each variable required to have at least one correlation with another variable above r = 0.3), and the Kaiser–Meyer–Olkin (KMO) measure needed to be at least 0.6, for sampling adequacy. In addition, Bartlett’s test of sphericity had to achieve statistical significance (p < 0.05). To establish components, the eigenvalue-one criterion was used. A Varimax orthogonal rotation was applied. A loading of 0.50 or greater was used to align items onto factors.

#### Cardiac synchrony

In order to record spectators’ synchrony in cardiac responses, before the beginning of the experimental session, three Ag/AgCl pre-gelled electrodes (ADInstruments, UK) with a contact area of 10 mm diameter were placed on participants’ skin in an Einthoven configuration to monitor their electrocardiogram (ECG). ECG was sampled at 1 kHz and online filtered with a Mains Filter (Powerlab and OctalBioAmp8/30, ADInstruments, UK). Participants’ ECG was recorded for the entire experimental phase. Two-minutes lasting time windows were chosen to appreciate the vagal and sympathetic components of heart rate variability associated with short-term cardiac modulation^17,52^. Baseline and rest periods (rest1 and rest2) were analyzed for their entire duration (2 minutes each). For consistency, the initial 2 minutes (T1) and the last 2 minutes (T2) of each monologue session were considered for the subsequent analyses. RR intervals were extracted detecting the R-peaks of each sequential heartbeat (i.e., tacogram) and then converted in RR time series (cubic spline interpolation with a re-sampling frequency rate at 4 Hz). For all participants and all conditions, each time series was 430 points in length. CRP MATLAB toolbox^53^ (available at http://www.agnld.uni-potsdam.de/~marwan/toolbox/) was used to compute the Cross Recurrence Plot (CRP)^54^ and the relative Cross Recurrence Quantification Analysis (CRQA)^55^. CRQA in-group metrics were extracted pairing each participant with the 3 spectators belonging to participant’s quartet. Adopting the same logic followed by previous studies^33,40,43,56^, out-group metrics were used as control measures. Indeed, synchrony comparisons across groups or conditions are commonly used benchmarks for synchrony specificity in the context of the investigated dyadic relationships. In our case, CRQA metrics were calculated pairing each participants with 3 randomly chosen spectators not belonging to the same quartet. If quartet’s synchronization will occur, in-group CRQA metrics must be significantly higher than out-group ones.

#### Cross recurrence plot (CRP)

CRP was developed to allow and facilitate the visualization of dynamical system recurrences in 2D. Briefly, CRP is an array of dots in a N × N square, where a dot is placed at (i, j) whenever x(j) is sufficiently close to x(i). To reconstruct the phase space, we used the time-delay (τ) method set at 1^57^. Embedding dimension (*m*) was estimated by the false nearest-neighbor method (FNN)^58^ for each time series in integer steps until the nearest neighbor becomes consistently less than 0.1. An optimal embedding dimension (*m*_*OPTg*_) was chosen as the highest *m* value among all time series in all conditions (m_OPTg_ = max {M(i,j)}). Our m_OPTg_ was set at 11. The last parameter defined was the Threshold (*ε*), which implements a cut-off limit that transforms the distance matrix into the recurrence matrix^57^. For each pair of time series, the selected threshold was the one giving the highest standard deviation in the identified recurrence states. Then, the optimal threshold used for the entire dataset was calculated as the mean value derived by the previously selected thresholds. Our optimal threshold was set at 0.2, coherently with the literature. The reason for keeping the threshold fixed was to allow comparison between different conditions.

#### Cross Recurrence Quantification Analysis (CRQA)

CRQA is a method developed to quantify the small-scale structures in recurrence plots^55^. Thanks to its implementation, CRQA allows quantification of the properties in the temporal evolution of a dynamic system, such as stability, complexity, and the occurrence of epochs of chaos vs. order. In the present study, four metrics were computed: Percentage recurrence (i.e., percentage of recurrent states), Entropy (i.e., complexity of the recurrent states), Laminarity (i.e., percentage of recurrent states forming vertical lines, which estimates the smoothness vs. chaoticity of the time series), and length of the longest diagonal line (i.e., maximum time during which the two time series were close to each other).

Functionally speaking, CRP and CRQA allow the visualization and the quantification of the overall density and time-consistency of recurrent cardiac responses to the same stimulus between people. Consequently, the four metrics computed different, but correlated, features of the same phenomenon providing a whole description of the synchrony pattern occurring between two people.

As stated above, since CRQA is a pairwise method, the 4 metrics were calculated for in-group and out-group pairs. The mean metrics of these pairs were taken as measure of individual in-group and out-group cardiac synchrony, respectively.

#### Correlations between behavioral measures and cardiac synchrony metrics

To test the hypothesis of a relation between in-group cardiac synchrony and the individual explicit ratings of monologues emotional intensity, for each participant we computed the in-group and out-group mean CRQA metrics measured during the enjoyment of both monologues and the individual mean explicit ratings about the emotional intensity of the two performances. Out-group mean CRQA metrics were included as a control for the specificity of the potential relation between in-group cardiac synchrony and individual mean explicit judgement. Pearson’s correlations (two-tails) were then performed between mean in-group CRQA metrics and the individual rating of emotional intensity. The critical probability value for multiple comparisons (n = 4) was corrected with Bonferroni method (p = 0.0125). The same procedure was followed for the out-group variables.

Proceeding from recent evidence about the spontaneous convergence in emotional intensity ratings between dyad members watching emotional movie clips together^59^, we were interested in investigating whether in-group cardiac synchrony could be related with in-group convergence in emotional intensity rating rather than with individual emotional intensity judgment, as hypothesized and tested before. Thus, for each participant we computed her/his in-group convergence score of emotional intensity rating. The in-group convergence score was calculated as the sum of the absolute values of the differences between participant’s absolute rating and the other three spectators’ absolute ratings. In-group convergence score was calculated for the two monologues and then averaged. High in-group convergence score corresponds to great participant’s deviation in the rating compared to spectators belonging to participant’s quartet. As control measure, out-group convergence score was also calculated. In this case, following the same pairing procedure adopted to extract out-group CRQA metrics, convergence score corresponds to the sum of the absolute values of the differences between participant’s absolute rating and the ratings of three random spectators’ not belonging to participant’s audience group.

Bonferroni corrected Pearson’s correlations were then performed between participants’ mean in-group CRQA metrics and their in-group convergence scores. Again, the critical probability value for multiple comparisons (n = 4) was corrected with Bonferroni method (p = 0.0125). The same analyses were then applied between participants’ mean out-group CRQA metrics and out-group convergence scores.

## Data Availability

All dependent variables or measures that were analyzed for this article’s target research question have been reported in the Methods section. All levels of all independent variables or all predictors or manipulations, whether successful or failed, have been reported in the Method section. The total number of excluded observations and the reasons for making those exclusions have been reported in the Method section. The raw data of this study are published on the following link https://data.mendeley.com/datasets/pnbbs7s736/draft?a=abca7be6-12e2-4418-82ba-fd3c42667b25.

## References

[1] Sosis R, Ruffle BJ (2004). Ideology, Religion, and the Evolution of Cooperation: Field Experiments on Israeli Kibbutzim. Res. Econ. Anthropol..

[2] Sullivan GB (2018). Collective emotions: A case study of South African pride, euphoria and unity in the context of the 2010 FIFA World Cup. Front. Psychol..

[3] Greenaway KH, Kalokerinos EK, Williams LA (2018). Context is Everything (in Emotion. Research). Soc. Personal. Psychol. Compass.

[4] Sutherland ME (2009). The influence of social situations on music listening. Ann. N. Y. Acad. Sci..

[5] Egermann H (2011). Does music listening in a social context alter experience? a physiological and psychological perspective on emotion. Music. Sci..

[6] Dunand M, Berkowitz L, Leyens J (1984). Audience effects when viewing aggressive movies. Br. J. Soc. Psychol..

[7] Kaltwasser L (2019). Sharing the filmic experience - The physiology of socio-emotional processes in the cinema. PLoS One.

[8] Kwon J, Ogawa KI, Ono E, Miyake Y (2015). Detection of nonverbal synchronization through phase difference in human communication. PLoS One.

[9] Shockley K, Santana MV, Fowler CA (2003). Mutual Interpersonal Postural Constraints are Involved in Cooperative Conversation. J. Exp. Psychol. Hum. Percept. Perform..

[10] Latif N, Barbosa AV, Vatiokiotis-Bateson E, Castelhano MS, Munhall KG (2014). Movement coordination during conversation. PLoS One.

[11] von Zimmermann J, Richardson DC (2016). Verbal synchrony and action dynamics in large groups. Front. Psychol..

[12] Kawasaki M, Yamada Y, Ushiku Y, Miyauchi E, Yamaguchi Y (2013). Inter-brain synchronization during coordination of speech rhythm in human-to-human social interaction. Sci. Rep..

[13] Codrons, E., Bernardi, N. F., Vandoni, M. & Bernardi, L. Spontaneous group synchronization of movements and respiratory rhythms. *PLoS One* **9** (2014). 10.1371/journal.pone.0107538 PMC4162643 25216280

[14] Müller, V. & Lindenberger, U. Cardiac and respiratory patterns synchronize between persons during choir singing. *PLoS One* **6** (2011). 10.1371/journal.pone.0024893 PMC3177845 21957466

[15] Néda Z, Ravasz E, Brechet Y, Vicsek T, Barabási A-L (2000). The sound of many hands clapping..

[16] Bernardi NF (2017). Increase in synchronization of autonomic rhythms between individuals when listening to music. Front. Physiol..

[17] Palumbo RV (2017). Interpersonal Autonomic Physiology: A Systematic Review of the Literature. Personal. Soc. Psychol. Rev..

[18] Golland, Y., Arzouan, Y. & Levit-Binnun, N. The mere Co-presence: Synchronization of autonomic signals and emotional responses across Co-present individuals not engaged in direct interaction. *PLoS One* **10**, 1–13 (2015). 10.1371/journal.pone.0125804 PMC4446307 26018597

[19] Launay J, Dean RT, Bailes F (2014). Synchronising movements with the sounds of a virtual partner enhances partner likeability. Cogn. Process..

[20] Lumsden J, Miles LK, Neil Macrae C (2014). Sync or sink? Interpersonal synchrony impacts self-esteem. Front. Psychol..

[21] Valdesolo P, Ouyang J, DeSteno D (2010). The rhythm of joint action: Synchrony promotes cooperative ability. J. Exp. Soc. Psychol..

[22] Chanel G, Kivikangas JM, Ravaja N (2012). Physiological compliance for social gaming analysis: Cooperative versus competitive play. Interact. Comput..

[23] Levenson RW, Ruef AM (1992). Empathy: A Physiological Substrate. J. Pers. Soc. Psychol..

[24] Slovák, P., Tennent, P., Reeves, S. & Fitzpatrick, G. Exploring skin conductance synchronisation in everyday interactions. In *Proceedings of the NordiCHI 2014: The 8th Nordic Conference on Human-Computer Interaction: Fun, Fast, Foundational* 511–520, 10.1145/2639189.2639206 (2014).

[25] Vacharkulksemsuk T, Fredrickson BL (2012). Strangers in sync: Achieving embodied rapport through shared movements. J Exp Soc Psychol..

[26] Walton AE (2018). Creating Time: Social Collaboration in Music Improvisation. Top. Cogn. Sci..

[27] Muszynski M, Kostoulas T, Lombardo P, Pun T, Chanel G (2018). Aesthetic highlight detection in movies based on synchronization of spectators’ reactions. ACM Trans. Multimed. Comput. Commun. Appl..

[28] Muszynski, M., Kostoulas, T., Lombardo, P., Pun, T. & Chanel, G. Synchronization among groups of spectators for highlight detection in movies. *Proc. 2016 ACM Multimed. Conf*. 292–296, 10.1145/2964284.2967229 (2016).

[29] McNeill, W. H. *Keeping together in Time*. *Harvard University Press*. (1995).

[30] Scherer KR (2005). What are emotions? and how can they be measured?. Soc. Sci. Inf..

[31] Ardizzi M (2014). When age matters: differences in facial mimicry and autonomic responses to peers’ emotions in teenagers and adults. PLoS One.

[32] Ardizzi M (2016). Less Empathic and More Reactive: The Different Impact of Childhood Maltreatment on Facial Mimicry and Vagal Regulation. PLoS One.

[33] Vicary S, Sperling M, von Zimmermann J, Richardson DC, Orgs G (2017). Joint action aesthetics. PLoS One.

[34] Wassiliwizky E, Koelsch S, Wagner V, Jacobsen T, Menninghaus W (2017). The emotional power of poetry: Neural circuitry, psychophysiology and compositional principles. Soc. Cogn. Affect. Neurosci..

[35] Siri F (2018). Behavioral and autonomic responses to real and digital reproductions of works of art. Prog. Brain Res..

[36] Richardson MJ, Marsh KL, Schmidt RC (2005). Effects of visual and verbal interaction on unintentional interpersonal coordination. J. Exp. Psychol. Hum. Percept. Perform..

[37] Richardson MJ, Marsh KL, Isenhower RW, Goodman JRL, Schmidt RC (2007). Rocking together: Dynamics of intentional and unintentional interpersonal coordination. Hum. Mov. Sci..

[38] Schmidt RC, Nie L, Franco A, Richardson MJ (2014). Bodily synchronization underlying joke telling. Front. Hum. Neurosci..

[39] Kinreich, S., Djalovski, A., Kraus, L., Louzoun, Y. & Feldman, R. Brain-to-Brain Synchrony during Naturalistic Social Interactions. *Sci. Rep.* **7**, 1–12 (2017). 10.1038/s41598-017-17339-5 PMC5719019 29213107

[40] Dikker S (2017). Brain-to-Brain Synchrony Tracks Real-World Dynamic Group Interactions in the Classroom. Curr. Biol..

[41] Swarbrick D (2019). How live music moves us: Head movement differences in audiences to live versus recorded music. Front. Psychol..

[42] Bachrach A, Fontbonne Y, Joufflineau C, Ulloa JL (2015). Audience entrainment during live contemporary dance performance: Physiological and cognitive measures. Front. Hum. Neurosci..

[43] Konvalinka I (2011). Synchronized arousal between performers and related spectators in a fire-walking ritual. Proc. Natl. Acad. Sci..

[44] Cela-Conde, C. J. & Ayala, F. J. *Art and brain coevolution*. *Progress in Brain Research* **237**, (Elsevier B.V., 2018). 10.1016/bs.pbr.2018.03.013 29779746

[45] Pelowski M, Markey PS, Forster M, Gerger G, Leder H (2017). Move me, astonish me… delight my eyes and brain: The Vienna Integrated Model of top-down and bottom-up processes in Art Perception (VIMAP) and corresponding affective, evaluative, and neurophysiological correlates. Phys. Life Rev..

[46] Ardizzi Martina, Ferroni F., Siri F., Umiltà M. A., Cotti A., Calbi M., Fadda E., Freedberg D., Gallese V. (2018). Beholders’ sensorimotor engagement enhances aesthetic rating of pictorial facial expressions of pain. Psychological Research.

[47] Jola C, Grosbras MH (2013). In the here and now: Enhanced motor corticospinal excitability in novices when watching live compared to video recorded dance. Cogn. Neurosci..

[48] Norèn, L. Dossier pédagogique Le 20 Novembre. *La Cie. La CamAra Oscura* (2008).

[49] Carver CS, White TL (1994). Behavioral Inhibition, Behavioral Activation, and Affective Responses to Impending Reward and Punishment: The BIS/BAS Scales. J. Pers. Soc. Psychol..

[50] Davis MH (1983). Measuring individual differences in empathy: Evidence for a multidimensional approach. J. Pers. Soc. Psychol..

[51] Bagby M, Parker JDA, Taylor GJ (1994). The twenty-item Toronto Alexithymia Scale-I. Item selection and cross-validation of the factor structure..

[52] Berntson GG (1997). Heart rate variability: origins, methods, and interpretive caveats. Psychophysiology.

[53] Marwan, N. *Reference Manual*. *Technology* **1** (2010).

[54] Eckmann JP, Kamphorst SO, Ruelle D (1987). Recurrence Plots of Dynamic System. Eur. Lett..

[55] Webber, C. L. J. & Zbilut, J. P. Recurrence Quantification Analysis of Nonlinear Dynamical Systems. in *Tutorials in contemporary nonlinear methods for the behavioral sciences* 26–94 (2005).

[56] von Zimmermann J, Vicary S, Sperling M, Orgs G, Richardson DC (2018). The Choreography of Group Affiliation. Top. Cogn. Sci..

[57] Zbilut JP, Giuliani A, Webber CL (1998). Detecting deterministic signals in exceptionally noisy environments using cross-recurrence quantification. Phys. Lett. A..

[58] Kennel MB, Brown R, Abarbanel HDI (1992). Determining embedding dimension for phase-space reconstruction using a geometrical construction. Phys. Rev. A..

[59] Bruder M, Dosmukhambetova D, Nerb J, Manstead ASR (2012). Emotional signals in nonverbal interaction: Dyadic facilitation and convergence in expressions, appraisals, and feelings. Cogn. Emot..

